# Annotations

**Published:** 1952-04

**Authors:** 


### *Annotations*

### PROBLEM FAMILIES IN BRISTOL

Since the war ended much attention has been directed to *problem families*, in which the children are neglected, the parents irresponsible and the home is squalid. Several recent surveys have attempted to estimate their number and some of their main characteristics. The Bristol survey in 1949 (Occasional Papers on Eugenics, No. 6, Eugenics Society, 1950) showed that there were probably 155 bad problem families in the city, nearly 60 per cent. of whom were living on Corporation housing estates. These families had a high average number of children (5·8), many of whom were subsequently removed from the parents and became a direct charge on the state. Illegitimacy, child neglect, and juvenile delinquency were common. Ill-health of the mother, subnormal intelligence of both parents and educational retardation in the children were also found to be characteristic. Many of the families were living in a state of poverty. These families rarely show any improvement in spite of all the assistance given to them by health visitors, sanitary inspectors, educational welfare officers and other medico-social workers.

Some Authorities think that such families should be isolated from the rest of the community and housed in special buildings where they can do least damage and be kept under close supervision while attempts are made to re-house them. This practice was tried in Holland and failed largely because the families became outcasts and lost ail self-esteem so that attempts at rehabilitation were doomed to failure. Torquay plans to build special flats for them and it will be interesting to see to what extent this experiment succeeds. Other authorities have recommended their segregation in camps where rehabilitation can be attempted. In Bristol this has happened unintentionally, for many of the families, unable to pay their rent, have been evicted, and the Welfare Authority has had no accommodation other than camps in which to re-house them. Now we have three camps largely occupied by problem families. Attempts so far made to reclaim them have proved fruitless and some other method will have to be tried.

During the war the *Pacifists Service Units* began work on these families in one or two large cities. Their volunteers were prepared to keep the families under very close supervision and control, giving them help in every possible way, *e.g.* by taking their children to school, seeing that the children attended school clinics, helping to have their houses disinfested and redecorated and assisting them in obtaining furniture and furnishings thereafter. The mothers were helped to plan the spending of their weekly allowance and every attempt was made to encourage self-help. The work of the Units has been continued since the war under the title of *Family Service Units*, largely at present operating in Liverpool, Salford and London. The Lancashire Community Council have also undertaken the training of the mothers of problem families at a recuperation centre but the results have not yet been published. This is not entirely satisfactory because only mothers and children are admitted, while the fathers remain at home, often untouched by any social agency.

After the Bristol inquiry the City Council took powers to employ home advisers after the style of the Family Service Units and to provide a recuperation centre, and furniture and furnishings free of charge or on loan. It is understood that enquiries are being made to see whether any voluntary organization is willing to undertake this work on their behalf.

This work is best done by volunteers for it requires a strong missionary spirit and a willingness to help at any time of the day or night. Also the absence of officialdom encourages the right atmosphere for rehabilitation.

### SOUTH WESTERN REGIONAL HOSPITAL EXPENDITURE IN 1952–53

It has recently been reported in the Press that £15,189,000 may be available for the hospital services in this region during the year 1952–53.

This is a large sum of money and after reading these Press reports many people imagine that all will be well in the hospital service during 1952–53 and money will cease to be a difficulty. The situation is not so favourable. Firstly the amount allocated is some £900,000 less than the Region really requires. Secondly, of the £15,189,000, the Ministry has earmarked a very large reserve to cover increases in salaries and wages. This reserve cannot be spent in any other way.

Officers of the Board have recently talked with Chairmen and Officers of the thirty-seven Hospital Management Committees. As a result, the Board has decided to allocate to the Hospital Management Committees the sum of £12,650,683 for running the hospital services during 1952–53 at the same level as they were on the 31st March, 1952. The amounts allocated to Hospital Management Committees have been made in the light of the information revealed by costing statements. Comparative costings of hospitals, classified size within type, were available to the Board for the first time since the inception of the Health Service.

The object of the regional allocation this year has been to try to maintain the hospital service during 1952–53 at the level which it attained in March, 1952, and it must be borne in mind that Management Committees have only been given an extra £361,000 to meet the rise in expenditure of items other than salaries and wages over the whole year. Everybody knows about the steady rise in prices of provisions, fuel, light and heat, etc., and Management Committees are going to be hard pressed to maintain their March, 1952, level throughout the whole of the next accounting year within the sum of £12,650,683.

It is true to say, however, that the hospital service cannot stand still—it must either progress or retrogress. In order to ensure some progress, however small, the Board has found the sum of £135,000, less than 1 per cent. of the total allocated to the region, to provide for development and extension of services during the year.

If, Management Committees can save out of the sum allocated to them, they will be able, with the approval of the Board, to use this money for further developments and improvements within their groups.

Owing to the shortage of labour and materials the Ministry has limited the amount to be spent on the maintenance of hospital premises, plant and grounds. The amount given to Hospital Management Committees for this is £920,497, and this sum is part of the total Hospital Management Committees' allocation of £12,650,683.

The cost of administration of the Board itself is £101,000 or 0·66 per cent. of the total allocated to the Region; the Blood Transfusion Service, Miniature Mass Radiography Service, Cancer Records Bureau, and Contractual arrangements with convalescent homes outside the Health Service account for £280,950; the provision for the Specialist and Registrar Service throughout the Region is £1,248,900; and the balance of the Board's expenditure covers the provision of long-stay annexes to mental Hospitals, of preventive medicine and pathological services in the Bristol Clinical Area, and medical research.

### UNITED BRISTOL HOSPITALS EXPENDITURE 1952–53

The Board of Governors has heard that £1,276,000 will be available for maintenance expenditure in 1952–53. Of this £56,000 is ear-marked as a special reserve against salary and wage awards and cannot be used for any other purpose. The net sum available is therefore £1,220,000. The Board had asked for £1,239,000 which included £14,000 for developments. It therefore appears that in 1952–53 the Board will have no money for developments and will have to make a further saving of £5,000. In the present state of the country's finances we know that it is useless to budget for developments, except those which inevitably arise as a direct consequence of decisions of policy to which we are already committed. One such example arises from the present excellent rate of recruitment of nurses to the Group. Every year greater numbers of them have to be housed, fed and clothed. The increase in their numbers permits an increase in work in the Wards and Departments such as can be noted in the following figures

| | 1949 | 1950 | 1951 |
|---|---|---|---|
| In-patients treated .. | 19,648 | 20,111 | 21,782 |
| Operations .. .. | 19,787 | 20,687 | 22,416 |

As the work increases more money is spent on drugs, dressings, medical and surgical appliances, fuel, light and power, and so on, but the money available does not increase proportionately. If the yearly increases in the cost of everything are added it becomes quite plain that our very modest demands for developments can be met only by economies elsewhere.

The Board faces this kind of problem every year and so far has made adjustments so that neither work nor developments suffer. The result is that by now most of the possible economy measures have been fully exploited and we have reached a stage where saving money generally implies a cut in one of the ancillary services, necessitating the resumption by professional and trained staff of a certain amount of work which would normally be performed by the unskilled.

### FAMILY PLANNING ASSOCIATION

The Bristol Branch, started in November 1951, has sessions at the Central Health Clinic, Tower Hill, Bristol 2 on Wednesdays at 6.30 p.m. The Branch is controlled by an executive committee of which Professor Gordon Lennon is the chairman. The doctor in charge (Dr. Constance Roberts), gives advice on family planning, marital problems, and contraceptive methods.

The fee for consultations is 5s. (it may be reduced in certain circumstances). This fee covers two or more visits within a period of six months. Appliances are paid for by the patient.

Beginning in March 1952 it is proposed to offer training to general practitioners and to nurses who are either S.R.N. or S.C.M. or have nursing experience which is considered adequate by the training committee.

Further details may be obtained from the Secretary.

